# Biology and interactions of two distinct monopartite begomoviruses and betasatellites associated with radish leaf curl disease in India

**DOI:** 10.1186/1743-422X-9-43

**Published:** 2012-02-16

**Authors:** AK Singh, B Chattopadhyay, S Chakraborty

**Affiliations:** 1 Molecular Virology Laboratory, School of Life Sciences, Jawaharlal Nehru University, New Delhi 110 067, India; 2 School of Life Sciences, Central University of Gujarat, Gandhinagar 382030, Gujarat, India

**Keywords:** Radish, Begomovirus, Leaf curl, Interaction, Novel species, India

## Abstract

**Background:**

Emerging whitefly transmitted begomoviruses are major pathogens of vegetable and fibre crops throughout the world, particularly in tropical and sub-tropical regions. Mutation, pseudorecombination and recombination are driving forces for the emergence and evolution of new crop-infecting begomoviruses. Leaf curl disease of field grown radish plants was noticed in Varanasi and Pataudi region of northern India. We have identified and characterized two distinct monopartite begomoviruses and associated beta satellite DNA causing leaf curl disease of radish (*Raphanus sativus*) in India.

**Results:**

We demonstrate that RaLCD is caused by a complex of two Old World begomoviruses and their associated betasatellites. Radish leaf curl virus-Varanasi is identified as a new recombinant species, *Radish leaf curl virus *(RaLCV) sharing maximum nucleotide identity of 87.7% with Tomato leaf curl Bangladesh virus-[Bangladesh:2] (Accession number AF188481) while the virus causing radish leaf curl disease-Pataudi is an isolate of Croton yellow vein mosaic virus-[India] (CYVMV-IN) (Accession number AJ507777) sharing 95.8% nucleotide identity. Further, RDP analysis revealed that the RaLCV has a hybrid genome, a putative recombinant between *Euphorbia leaf curl virus *and *Papaya leaf curl virus*. Cloned DNA of either RaLCV or CYVMV induced mild leaf curl symptoms in radish plants. However, when these clones (RaLCV or CYVMV) were individually co-inoculated with their associated cloned DNA betasatellite, symptom severity and viral DNA levels were increased in radish plants and induced typical RaLCD symptoms. To further extend these studies, we carried out an investigation of the interaction of these radish-infecting begomoviruses and their associated satellite, with two tomato infecting begomoviruses (*Tomato leaf curl Gujarat virus *and *Tomato leaf curl New Delhi virus*). Both of the tomato-infecting begomoviruses showed a contrasting and differential interaction with DNA satellites, not only in the capacity to interact with these molecules but also in the modulation of symptom phenotypes by the satellites.

**Conclusion:**

This is the first report and experimental demonstration of Koch's postulate for begomoviruses associated with radish leaf curl disease. Further observations also provide direct evidence of lateral movement of weed infecting begomovirus in the cultivated crops and the present study also suggests that the exchange of betasatellites with other begomoviruses would create a new disease complex posing a serious threat to crop production.

## Background

Geminiviruses (family *Geminiviridae*) have circular single stranded DNA genomes that are encapsidated in twinned quasi-isometric particles and are classified into four genera: *Begomovirus, Mastrevirus, Curtovirus *and *Topocuvirus *[1,2]. The largest genus, *Begomovirus *comprise of viruses transmitted by whitefly (*Bemisia tabaci *Genn.). Begomoviruses are assumed to have been co-evolving with their hosts for a long time, however, it is in the past two decades, these viruses have become an economically important plant pathogens [3-5]. These viruses contain genomes consisting of either one or two similar-size DNA components [6]. The DNA-A component encodes all viral functions required for replication, control of gene expression, encapsidation and vector transmission [7,8]. The DNA-B, code for two proteins which are involved in movement of the virus between and within plant cells [6]. Both components of bipartite begomoviruses are required for systemic infection and symptom induction [9]. In contrast, monopartite begomoviruses such as isolates of *Tomato yellow leaf curl virus, Tomato leaf curl virus *and *Cotton leaf curl Multan virus *[10-13] possess only a single genomic component resembling DNA-A, which alone is capable of inducing disease symptoms. However, for some other monopartite begomoviruses like *Ageratum yellow vein virus, Bhendi yellow vein mosaic virus, Cotton leaf curl Multan virus, Eupatorium yellow vein virus, Tomato yellow leaf curl China virus *and *Cotton leaf curl Gezira virus*, association of betasatellite (DNA-β) has been found to be essential for the induction of typical disease symptoms [14-19]. Betasatellite is a circular, single-stranded DNA molecule of ~1.35 kb length with a single open-reading frame (ORF) βC1, an adenine-rich region, a satellite conserved region having nonanulceotides (TAATATTAC) and it shares negligible sequence similarity with either DNA-A or DNA-B of bipartite begomoviruses [20-22].

Radish (*Raphanus sativus *L.; Family-*Brassicaceae*) is an important vegetable crop grown throughout India. Leaf curl disease of radish (RaLCD), for the first time, was reported from Punjab province of Pakistan [23]. In India, RaLCD was first reported from a homestead garden and adjoining farmer's field near Varanasi [24]. Later, RaLCD was also observed in Pataudi, Haryana, India. The infected plants remain stunted and leaves exhibited both upward and downward curling along with conspicuous vein enations.

In the present study, we have characterized two distinct begomoviruses and betasatellites associated with plants showing symptoms of RaLCD in Varanasi and Pataudi, India. These begomovirus species, together with the betasatellite can induce RaLCD. We further demonstrate the complexity of the interactions between RaLCD-associated satellites with radish and tomato-infecting begomoviruses indicating co-adaptation of weed infecting begomovirus in cultivated crop species like radish, hitherto a non-host.

## Results

### Cloning and genome organization of two begomovirus species associated with RaLCD in India

RaLCD symptoms developed on grafted *Raphanus sativus *plants were identical to those of naturally infected plants. The initial symptoms appeared as downward curling on the young leaves about 3-4 weeks after grafting, later infected leaves exhibited typical upward and downward leaf curling, enation on adaxial side and stunted growth of plants, which also failed to bear any flower. Southern blot hybridization and PCR based detection using begomoviruses specific primers [25] revealed association of begomovirus with RaLCD infected plants from Varanasi, Uttar Pradesh and Pataudi, Haryana in India. Full-length (~2.7 kb) viral genome from both samples (Cholapur and Pataudi) were cloned. Several putative full-length clones were obtained and preliminary restriction analysis indicated the presence of single class of molecule in both samples (data not shown). Two full-length molecules from each sample were selected and sequenced. The complete sequence of RaLCD-associated-Varanasi virus (GenBank EF175733) and RaLCD-associated-Pataudi virus (GenBank FJ593629) isolate were of 2756 nucleotides (nt) and 2759 nt, respectively. The genome organization of both the isolates was similar to that of Old World monopartite begomoviruses [i.e. two virus-sense ORFs (V1 and V2) and four complementary-sense ORFs (C1, C2, C3, C4)]. Intergenic region (IR) sequences (~290 nt) contained conserved nonanucleotide sequence, putative Rep (C1) protein binding sites and the TATA box. The nucleotide identity of full-length genome and IR sequences between RaLCD-associated-Varanasi and RaLCD-associated-Pataudi were 79.6 and 71.6%, respectively, indicating involvement of two distinct species (Table 1).

**Table 1:** Nucleotide identities (%) for full-length genome (nt) and IR and amino acid (aa) sequence identities (%) for ORFs of two begomoviruses associated with RaLCD in India and selected previously characterized begomoviruses

Begomoviruses	DNA-A	Rep	TrAP	REn	AC4	CP	AV2	ICR
**RaLCV-[IN:Var:05]**								

ToLCBDV-[BD:2]	**87.7**	**84.8**	**80.6**	**86.6**	54.6	**94.1**	**98.8**	75.3

TbCSV-[CN:Yn35:01]	**87.0**	**84.5**	**87.3**	**88.1**	50.5	**93.9**	**99.2**	71.2

AEV-[NP:01]	**86.9**	**84.5**	**85.8**	**88.1**	65.9	**93.9**	**98.4**	74.0

TbCSV-[CN:Yn282:Age:03]	**86.9**	**83.9**	**88.1**	**88.1**	48.0	**93.0**	**99.2**	71.2

PaLCuV-IN[IN:Luc]	**86.4**	73.6	**96.3**	**97.0**	44.7	**91.5**	**97.7**	58.0

CLCuKV-Man[PK:M806b:96]	**85.8**	**87.5**	**84.3**	**82.8**	66.0	**96.6**	**98.8**	40.2

EuLCV-[CN:Gx35:02]	**85.7**	**90.0**	**82.1**	**88.1**	**86.5**	78.0	**93.8**	69.7

ChiLCV-IN[IN::05]	**84.0**	**86.4**	**82.1**	73.1	52.9	**98.8**	**89.3**	75.3

ChiLCV-Mul[PK:Mul:98]	**83.0**	**84.5**	79.4	**80.6**	53.6	**87.3**	**96.1**	64.4

ToLCGV-[IN:Var:01]	**80.7**	**83.1**	79.9	77.6	51.0	**86.1**	**80.9**	68.4

CYVMV-[IN]	**80.4**	75.9	**83.6**	**87.3**	48.2	**87.3**	83.2	59.1

BYVMV-IN[IN:Mad]	74.9	**81.2**	64.9	67.9	69.6	70.3	**93.4**	38.7

ToLCNDV-IN[IN:ND:Svr:92]	74.2	77.8	60.4	66.4	62.1	73.2	**93.0**	60.0

SLCCNV-IN[IN:Luc:Pum]	73.6	75.6	57.5	64.8	53.5	71.4	**93.0**	40.3

MYMIV-[IN:ND:Bg3:91]	63.5	70.6	52.2	40.2	45.5	50.0	72.2	10.9

**CYVMV-[IN:Pat:Rad:08]**								

RaLCV	79.6	75.6	**82.8**	**89.6**	44.7	**83.2**	**87.3**	71.6

ToLCBDV-[BD:2]	**80.1**	77.0	**85.8**	**84.3**	54.6	**82.8**	**91.5**	67.5

TbCSV-[CN:Yn35:01]	80.0	76.7	77.6	**85.1**	35.3	**83.2**	**92.2**	71.3

AEV-[NP:01]	**80.9**	77.8	79.9	**86.6**	52.9	**82.4**	**91.3**	70.5

TbCSV-[CN:Yn282:Age:03]	79.9	76.2	78.4	**85.1**	34.1	**83.2**	**91.3**	71.3

PaLCuV-IN[IN:Luc]	**86.0**	**92.2**	**81.3**	**88.1**	**95.3**	**82.4**	**97.7**	59.7

CLCuKV-Man[PK:M806b:96]	78.1	75.0	**86.6**	**87.3**	44.7	**83.6**	**98.8**	54.5

EuLCV-[CN:Gx35:02]	78.1	77.7	**85.8**	**88.8**	45.9	79.7	72.9	73.3

ChiLCV-IN[IN::05]	78.4	77.3	**82.1**	77.6	35.3	**82.4**	**89.0**	70.1

ChiLCV-Mul[PK:Mul:98]	78.3	76.5	**82.4**	**84.3**	31.8	**82.0**	**83.1**	74.6

ToLCGV-[IN:Var:01]	**80.5**	76.5	**83.6**	**81.3**	35.3	**96.5**	**85.2**	66.9

CYVMV-[IN]	**95.8**	**94.7**	**96.3**	**93.3**	**90.6**	**100**	**98.3**	**94.4**

BYVMV-IN[IN:Mad]	71.3	70.0	64.2	71.6	40.0	80.5	66.9	54.2

ToLCNDV-IN[IN:ND:Svr:92]	71.5	69.5	64.9	64.2	34.5	82.8	68.8	64.2

SLCCNV-IN[IN:Var:Pum]	70.8	67.3	60.4	68.0	37.9	81.2	67.0	61.5

MYMIV-[IN:ND:Bg3:91]	62.0	65.8	50.0	40.2	30.6	74.1	47.3	46.2

*Identity over 80% is Bold

**Figure 1:**
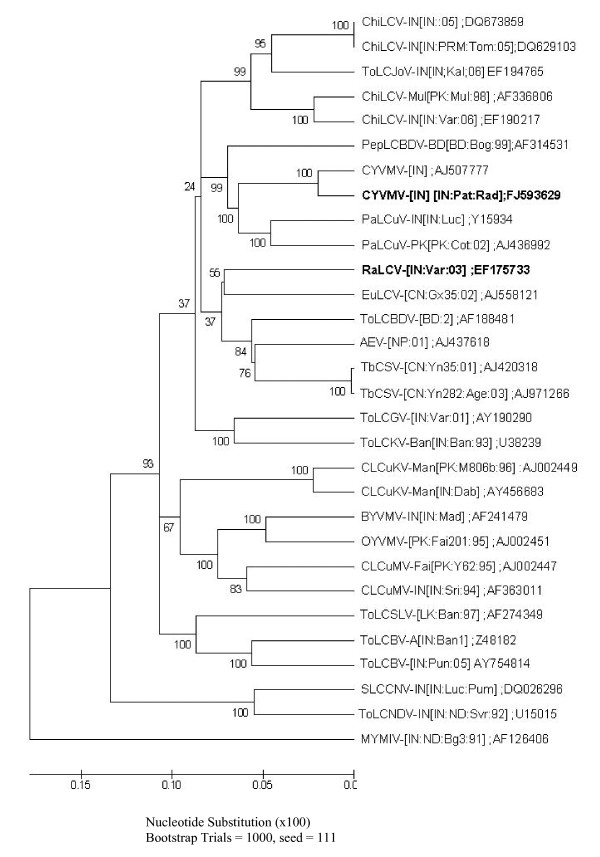
**Phylogenetic relationship of full-length DNA-A of radish-infecting begomoviruses with other begomoviruses**. Phylogenetic tree was generetaed based on aligned complete DNA-A sequences using ClustalW. All the sequences were obtained from GenBank. The viruses understudy are indicated as bold.

The sequence of the begomovirus DNA component of the RaLCD-associated-Varanasi virus had maximum nucleotide identity with Tomato leaf curl Bangladesh virus-[Bangladesh:2] (ToLCBDV-[BD:2]) (~87.7%) (Table 1). Phylogenetic analysis also support that RaLCD-associated-Varanasi is a distinct species, having very low bootstrap values with the Euphorbia leaf curl virus-[China:Guangxi 35:2002] (EuLCV-[CN:Gx35:02]) (Figure 1). Thus, RaLCD-associated-Varanasi virus is an isolate of a new begomovirus species, and the name Radish leaf curl virus-[India:Varanasi:2005] (RaLCV-[IN:Var:05]) is given following (ICTV) guidelines [2].

However, RaLCD-associated-Pataudi virus sequence was 95.8% identical to Croton yellow vein mosaic virus-[India] (CYVMV-[IN]) isolates (Table 1) and therefore, it can be considered as a variant of CYVMV-[IN] and designated as Croton yellow vein mosaic virus-[India:Pataudi:Radish:2008].

The predicted amino acid sequences of the ORFs of RaLCV-[IN:Var:05] were compared to those of other begomoviruses. TrAP, REn, CP and AV2 of RaLCV-[IN:Var:05] showed maximum amino acid identity (91-98%; Table 1) with Papaya leaf curl virus-India [India:Lucknow] (PaLCuV-IN[IN:Luc]) with which full-length DNA-A shared 86.4% identity (Table 1). However, AC4 and Rep shared maximum amino acid (aa) identity (86.5% and 90%, respectively) (Table 1) with weed-infecting begomovirus, EuLCV-[CN:Gx35:02]. Interestingly, IR of DNA-A showed maximum identity with ToLCBDV-[BD:2] (75.3%) (Table 1). Such divergence in the amino acid sequence of the encoded proteins present strong evidence of recombination in the genome of RaLCV-[IN:Var:05]. Sequences of the CYVMV-[IN:Pat:Rad:08] V1 (CP), V2, C1 (Rep), C2, C3 and IR ORFs were generally > 95% [nt and aa] identical with CYVMV-[IN], whereas maximum identity of C4 sequences (90% nt and 85% aa) were with PaLCuV-IN[IN:Luc] (Table 1).

A multiple alignment of the IR of six begomoviruses revealed that RaLCV-[IN:Var:05] and ToLCBDV-[BD:2] share very related iterons sequence (GGTGA-AC/T-GGTAC) whereas CYVMV-[IN:Rad:Pat:08] and CYVMV-IN iterons sequences were identical (GGGGA-CTC-GGGGGA) (data not shown). The major difference between the RaLCV-[IN:Var:05] and ToLCBDV-[BD:2] was found in spacer sequence of two iterons, as RaLCV-[IN:Var:05] contains AC while ToLCBDV-[BD:2] contains AT. A third iteron was present in 5' end of the intergenic common region (ICR) of RaLCV. Noticeably, besides GC rich region, stem loop region and the TATA Box were conserved for all geminiviruses and all the viruses tested here shared two short sequences, AATGGCA and TAAT, located at the 5'end of the region between TATA box and stem-loop.

### Cloning of betasatellite DNAs and genome organization

**Figure 2:**
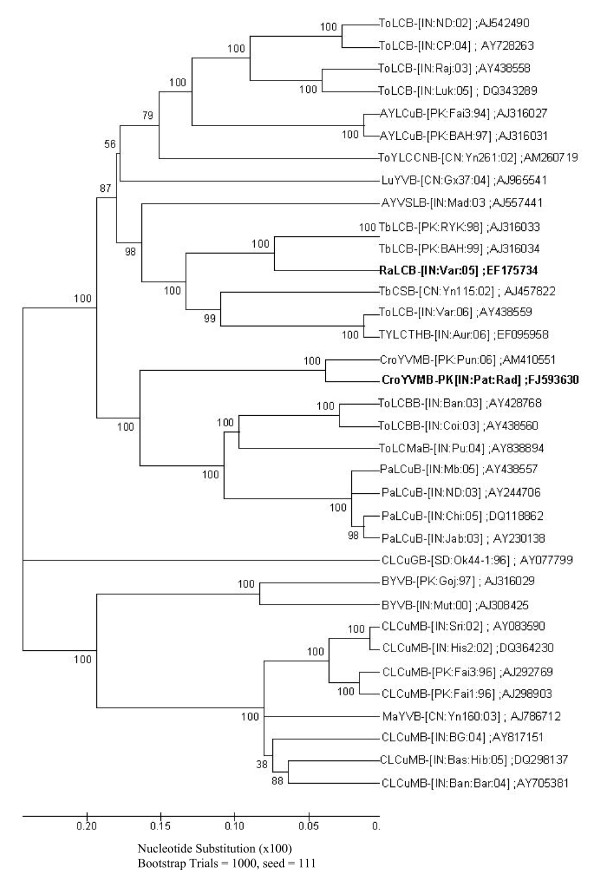
**Phylogenetic relationship of RaLCB and CroYVMB with betasatellites associated with other begomoviruses**. Phylogenetic tree was geenerated based on alignment of complete genome sequences using ClustalW. The viruses understudy are indicated as bold.

Despite repeated attempts, we could not identify any DNA-B molecule from the infected samples, indicating monopartite nature of virus isolates. Full-length genome (~1.3 kb) of betasatellites was cloned from both (Varanasi and Pataudi) RaLCD samples using universal primers [26]. The complete nucleotide sequence of betasatellites associated with RaLCV-[IN:Var:05] and CYVMV-[IN:Pat:Rad:08] was determined as 1358 nt (GenBank Accession no EF175734) and 1367 nt (GenBank Accession no. FJ593630), respectively. These two betasatellites had structural features similar to those of other betasatellites i.e. a single ORF βC1, satellite conserved region (SCR) and A rich region. The complete nucleotide sequence identity between RaLCV-[IN:Var:05] and CYVMV-[IN:Pat:Rad:08] was 63.2%, indicating that these are distinct betasatellite species. The complete sequence of DNA betasatellite associated with RaLCV-[IN:Var:05] and CYVMV-[IN:Pat:Rad:08] shared maximum nucleotide identity with Tobacco leaf curl betasatellite [Pakistan:Rahim Yar Khan:1998] (TbLCB-[PK:RYK:98]) (83%) and Croton yellow vein mosaic betasatellite [Pakistan:Punjab:2006] (CroYVMB-[PK:Pun:06]) (91.1%), respectively (Table 2). Phylogenetically, RaLCV-[IN:Var:05] associated DNA betasatellite clusterd with the one associated with tobacco disease with TbLCB-[PK:RYK:98], whereas the CYVMV-[IN:Pat:Rad:08] associated DNA betasatellite clustered with another isolate of this species. (Figure 2).

**Table 2:** Per cent identity (nucleotide) among DNA-β of two Radish leaf curl virus isolates and with selected begomoviruses originating in Asia

Beta	Percent	C1	SCR
TbLCB- Radish			

TbLCB-[PK:RYK:98]	**83.0**	**89.1(81.1)**	**84.4**

ToLCB-[IN:Var:06]	72.7	79.3(76.3)	**93.2**

TYLCTHB-[IN:Aur:06]	72.3	76.6(75.0)	**93.0**

AYVSLB-[IN:Mad:03]	68.2	75.9(71.2)	**90.0**

TbCSB-[CN:Yn115:02]	67.2	68.2(68.6)	**93.9**

ToYLCCNB-[CN:Yn261:02]	66.2	69.6(61.9)	**90.8**

AYLCuB-[PK:Fai3:94]	66.0	72.6(56.5)	**94.3**

CroYVMB-[PK:Pun:06]	62.5	62.6(57.5)	**91.7**

ToLCB-[IN:ND:02	62.0	72.1(66.9)	**80.3**

PaLCuB-[IN:Chi:05]	61.3	64.5(59.8)	**85.5**

PaLCuB-[IN:Jab:03]	61.2	65.7(61.9)	64.4

ToLCB-[IN:CP:04]	61.1	70.8(66.9)	**84.1**

**CYVMB-Radish**			

RaLCB-Var	63.2	64.7 (61.9)	**90.8**

TbLCB-[PK:RYK:98]	60.4	65.0 (61.0)	**83.8**

ToLCB-[IN:Var:06]	62.4	64.7 (57.6)	**91.9**

TYLCTHB-[IN:Aur:06]	61.8	65.0 (56.8)	**90.8**

AYVSLB-[IN:Mad:03]	59.7	66.7 (60.2)	**88.2**

TbCSB-[CN:Yn115:02]	59.8	63.3 (57.6)	**91.2**

ToYLCCNB-[CN:Yn261:02]	60.0	64.7 (60.2)	**86.4**

AYLCuB-[PK:Fai3:94]	62.3	66.0 (62.3)	**90.8**

CroYVMB-[PK:Pun:06]	**91.1**	**93.8 (90.7)**	**95.6**

ToLCB-[IN:ND:02]	59.8	65.8 (61.0)	**85.9**

PaLCuB-[IN:Chi:05]	60.8	70.9 (66.9)	**87.6**

PaLCuB-[IN:Jab:03]	61.3	71.4 (66.1)	**84.1**

ToLCB-[IN:CP:04]	59.8	63.9 (61.0)	**85.8**

Figures in bracket indicate putative deduced amino acid sequence identity. *Identity over 80% is Bold

ORF βC1 of RaLCB-[IN:Var:05] and CYVMB-[IN:Pat:Rad:08] showed maximum nucleotide identity with TbLCB-Pak-[NIB 12-1] (89.1%) and CroYVMB-[PK:Pun:06] (93.8%), respectively (Table 2). Similarly, ORF βC1 encoded protein of RaLCV-[IN:Var:05] and CroYVMB-[IN:Pat:Rad:08] associated DNA betasatellite showed maximum identity with TbLCB-Pak-[NIB 12-1] and CroYVMB-[PK:Pun:06] (87.3% and 90.7%, respectively) (Table 2). The conserved ORF βC1 of RaLCB-[IN:Var:05] and CroYVMB-[IN:Pat:Rad:08] encoded a 13.7 kDa protein. The nucleotide sequences of SCR of RaLCV-[IN:Var:05] and CYVMV-[IN:Pat:Rad:08] associated DNA betasatellite showed > 90% identity with those of other DNA betasatellites (Table 2). SCR of RaLCV-[IN:Var:05] associated DNA betasatellite and CYVMV-[IN:Pat:Rad:08] associated DNA betasatellite showed maximum identity with AYLCuB (94.3%) and CroYVMB-[PK:Pun:06] (95.6%), respectively (Table 2).

### Detection of recombination

**Figure 3:**
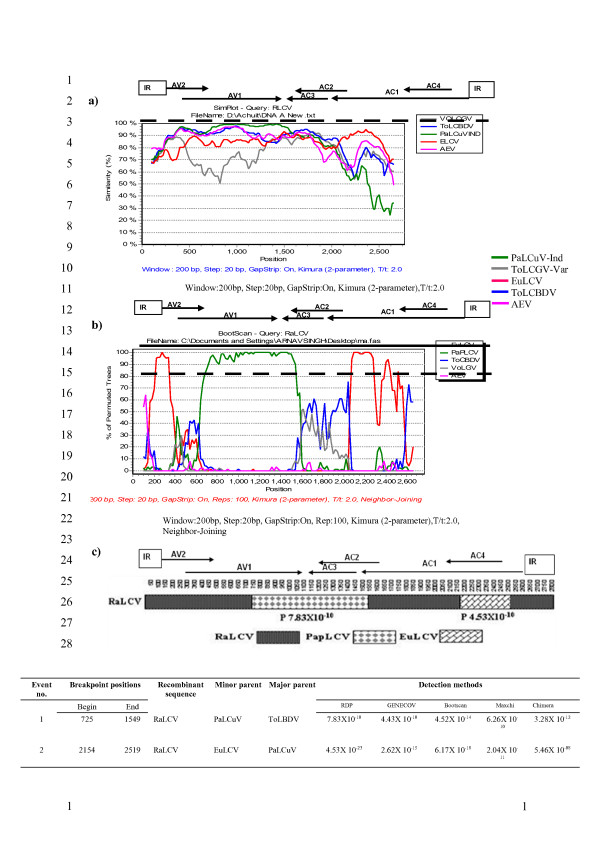
**Recombination analysis of RaLCV genome**. Similarity plot (**a**) and bootscanning (**b**) analyses of the RaLCV DNA-A using the SimPlot, version 2.5. Bootscan graph of RaLCV versus begomovirus strains constructed by using the neighbor-joining tree algorithm, the Kimura-2 distance model, and 100 pseudoreplicates. The dotted line shows an arbitrary 70% reliability threshold and the solid line shows the demarcation of species and strains (**c**) schematic representation and analysis of the linearized genomic DNAs of RaLCV showing the origin of the fragments, recombination breakpoints and putative parental viruses.

RaLCV-[IN:Var:05] (RLA) and CYVMV-[IN:Pat:Rad:08] (CRA) genomes were analyzed using two independent methods (simplot graph, and RDP analysis) and the results indicated definite evidence of recombination within RaLCV-[IN:Var:05] genome (Figure 3). In RaLCV-[IN:Var:05], two significant breakpoints detected at nts 725 and 1549 with significant 'P value' (0.01) (Figure 3c), corresponding to the region of the genome with > 90% identity with the sequence of PaLCuV. The N-terminal amino acid sequence of Rep was most similar to EuLCV (87%) than other geminiviruses. Evidence of recombination with EuLCV like virus was found within *Rep *coding region (from nt 2154 to 2519). Bootscan and Simplot analysis also supported these observations (Figure 3a, b). To confirm recombinant nature of RaLCV-[IN:Var:05] genome, dendrograms were also generated based on nucleotide sequence of virion sense ORF (AV1), complimentary sense ORFs (AC1, AC2 and AC3) with that of related begomoviruses. Interestingly, RaLCV-[IN:Var:05] clustered with PaLCuV when AV1, AC2 and AC3 sequences were used for phylogenetic analysis, whereas RaLCV-[IN:Var:05] was placed with EuLCV in the same clade in the dendrogram generated using AC1 nucleotide sequences (data not shown).

To detect possible evidence of recombination in the CYVMV-[IN:Pat:Rad:08] genome, Simplot and RDP analysis were performed with begomoviruse bearing the closest identity viz., CYVMV-[IN], and with two tomato-infecting begomoviruses viz., Tomato leaf curl Gujarat virus-[India:Varanasi:2001] (ToLCGV-[IN:Var:01]) and Tomato leaf curl New Delhi virus-India [India:New Delhi:Severe:1992] (ToLCNDV- [IN:ND:Svr:92]). Simplot and RDP analyses revealed that this is not a recombinant virus; rather it is a derivative of CYVMV-[IN]. Similarly, simplot and RDP analysis carried out for betasatellite molecules DNAs (RaLCB-[IN:Var:05] and CroYVMB-[IN:Pat:Rad:08]) could not reveal any evidence of recombination.

### Infectivity of cloned DNAs

**Figure 4:**
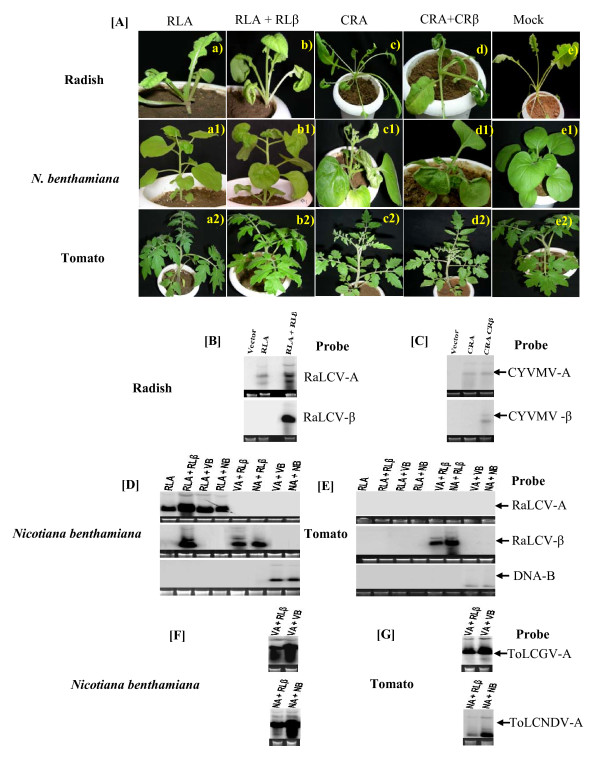
**Symptom expression and DNA accumulation in test plants**. Plant phenotypes [**A**] radish, *N. benthamiana *and tomato seedling following inoculation with a RaLCV DNA-A (RLA) and CYVMV-[IN:Pat:Rad:08] DNA-A (CRA)were inoculated either alone or with RaLCV DNA-betasatellites (RLβ) and CYVMV-[IN:Pat:Rad:08] betasatellites (CRβ). The photographs were taken at 30 dpi and southern blot analysis showing relative accumulation of viral DNA in inoculated [**B-C**] radish, [**D**] *N. benthamiana *(left panel)and [**E**] tomato (right panel) plants.

Partial tandem repeats of DNA-A and DNA betasatellite were constructed in binary vector and their infectivity were tested (either alone or together in combination) onto radish, tomato and *N. benthamiana *plants by agroinoculation. Radish plants co-inoculated with RLA + RLβ developed systemic symptoms including downward leaf curling within 20 days post inoculation (dpi) (Figure 4a, b; Table 3), however, RLA inoculated plants exhibited milder and delayed (6 days) symptoms as compared to infection by RLA + RLβ (Figure 4a; Table 3). Similarly, radish plants co-inocluated with CRA + CRβ exhibited severe leaf curling, twisting of petiole and enations symptoms (Figure 4a, d; Table 3), while CRA inoculated plants showed delayed (7 days) and milder symptom (Figure 4Ac; Table 3). The symptoms observed under experimental studies on radish plants were very similar to those observed under field conditions. The presence of viral genomic DNA and DNA betasatellites in infected radish plants was confirmed by Southern blot (Figure 4b and 4c) and PCR analysis (results not shown).

**Table 3:** Infectivity of viral genome and DNA betasatellites on radish plants

Test plant(s)	Construct combination used for agroinoculation	Plants showed symptom/Plants inoculated	Symptom	First symptom appearance (dpi)
Radish	RLA	11/20	MLC	26
	
	RLA + RLβ	15/20	SL, LC, Ens	20
	
	CRA	12/20	MLC,LR	24
	
	CRA+CRβ	17/20	LC, St, Ens,Tw	17
	
	VA+VB	0/16		
	
	NA+NB	0/16		

RLA, RaLCV; RLβ, RaLCB; CRA, CYVMV; CRβ, CroYVMB; NA, ToLCNDV DNA-A; NB, ToLCNDV DNA-B; VA ToLCGV-DNA-A; VB, ToLCGV-DNA-B; *MLC *mild leaf curling; *SL *small leaves; *LC *leaf curling; *Ens *enation; *LR *Leaf rolling; *Tw *twisting of petiole

**Figure 5:**
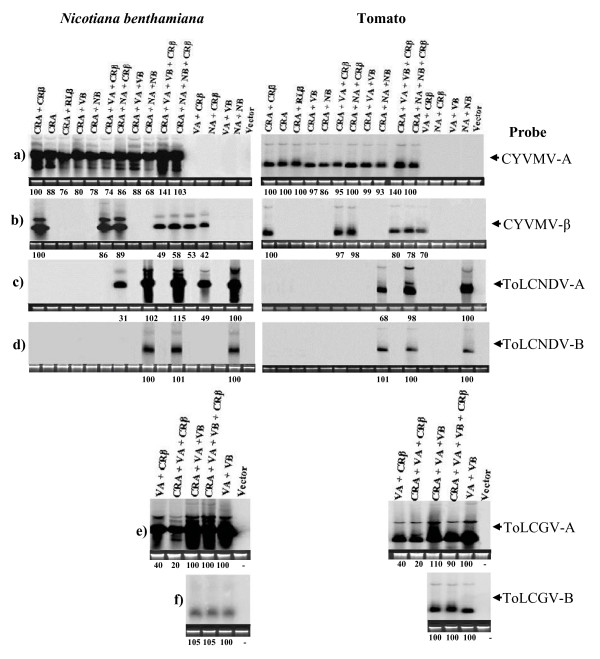
**Relative level of DNA accumulation following reassortment and mixed infection of begomoviruses**. Southern blot analysis showing relative accumulation of viral DNA and at the bottom of gel the numerical value is showing in percentage of viral DNA accumulation in inoculated tomato (right panel) and *N. benthamiana *(left panel).

Tomato plants agroinoculated with the RLA and RLβ did not develop any symptom (Figure 4a, b), whereas those co-inoculated with CRA and CRβ developed severe leaf curling and stunting symptoms. CRA infected plants produced systemic leaf curling on upper leaves and mild stunting at later stages (Figure 4;able 4), but when plants were co-inoculated with CRA and CRβ they were severely stunted (Figure 4; Table 4). Agroinoculation of infectious clones of RLA and CRA to *N. benthamiana *resulted in leaf curling, enations and mild stunting of test plants (Figure 4Aa1 and 4Ac1) followed by typical severe leaf curling, twisting of petiole, interveinal chlorosis, severe stunting and enations on underside of leaves when co-inoculated with RLβ and CRβ, respectively (Figure 4Ab1 and 4Ad1; Table 4). Presence of viral genomic DNA and DNAβs in infected *N. benthamiana *and tomato were confirmed by Southern blot (Figures 4D, E, 5a and 5b) and PCR analysis (results not shown). Accumulation of DNA component (RLA and CRA) was enhanced when DNA betastaellite was associated.

**Table 4:** Constructs, their infectivity and symptoms induced on agroinoculated plants

Test plant(s)	Construct combination used for agroinoculation	Plants showed symptom/Plants inoculated	Symptom	First symptom appearance (dpi)
*N. benthamiana*	RLA	7/16	LC, Ens	20
	
	RLA + RLβ	12/16	LC, Ens, IVCL, Tw, Bls	8
	
	RLA+VB	5/16	LC, Ens	20
	
	RLA+NB	5/16	LC, Ens	20
	
	VA+RLβ	16/16	LC, IVCL, CR, LD, St, BLs, SL	5
	
	NA+RLβ	16/16	LC, IVCL, CR, St, BLs, SL	7

Tomato	RLA	0/16	-	-
	
	RLA + RLβ	0/12	-	-
	
	RLA+VB	0/12	-	-
	
	RLA+NB	0/12	-	-
	
	VA+RLβ	12/12	SLC, CR, LD, St, BLs, SL, LPk	8
	
	NA+RLβ	7/12	LC, CR, YL, Tw	18

*N. benthamiana*	CRA	16/16	LC, Ens, Tw	7
	
	CRA+CRβ	16/16	LC, Ens, IVCL, Bls, St, SL	6
	
	CRA+VB	16/16	LC, Ens, Tw	7
	
	CRA+NB	16/16	LC, Ens, Tw	7
	
	CRA+ RLβ	16/16	LC, Ens, IVCL, Bls, St, SL	6
	
	VA+CRβ	16/16	LC, IVCL, BLs, Tw	6
	
	NA+CRβ	16/16	LC, MY, VC, Bls,	6
	
	CRA+VA+CRβ	16/16	LC, IVCL, CR, Ens, St, BLs, Tw	5
	
	CRA+NA+CRβ	16/16	LC, Bls, IVCL, Ens, Tw	5
	
	CRA+VA+VB	16/16	LC, IVCL, CR, Ens, Chls, BLs, SL, Tw	5
	
	CRA+NA+NB	16/16	LC, YL, VB, St, Mt, Bls, IVCL, Ens	5
	
	CRA+VA+VB+β	16/16	LC, IVCL, Ens, LD, St, Bls, SL, Tw	5
	
	CRA+NA+NB+β	16/16	SL, YL, St, LC, Mt, Tw	5
	
	VA+VB	10/10	LC, IVCL, CR, LD, Chls, Bls, SL	6
	
	NA+NB	10/10	SL, YL, LC, Mt	5

Tomato	CRA	10/12	LC, LR	21
	
	CRA+CRβ	12/12	LC, LR, VC, SL	18
	
	CRA+VB	10/12	LC, LR	21
	
	CRA+NB	9/12	LC, LR	21
	
	CRA+ RLβ	12/12	LC, LR, VC, SL	19
	
	NA+CRβ	0/12	-	-
	
	VA+CRβ	10/12	LC, MY	9
	
	CRA+VA+CRβ	12/12	LC, LR, VC, SL, MY	9
	
	CRA+NA+CRβ	12/12	LC, LR, VC, SL	16
	
	CRA+VA+VB	12/12	LC, LR, CR, YL	7
	
	CRA+NA+NB	12/12	LC, LR, VC, SL, YL	8
	
	CRA+VA+VB+β	12/12	LC, LR, CR, YL, St	7
	
	CRA+NA+NB+β	12/12	LC, LR, VC, SL, YL	8
	
	VA+VB	10/10	LC, CR, LR, YL	7
	
	NA+NB	10/10	LC, CR, YL	8

*LC *leaf curling; *BV *big veins; *MY *mild yellowing; *YL *yellow leaf lamina; *St *stunting; *LD *leaf distortion; *VB *vein banding; *MO *severe mosaic; *LR *leaf rolling; *Bls *blistering; *SL *small leaves; *CR *leaf crinkling; *IVCL *interveinal chlorosis; *Chls *chlorotic spots; *MiMO *mild mosaic; *Mt *mottling; *SYL *severe yellow leaf lamina; *Tw *twisting of petiole *VC *vein clearing, *LPk *Leaf puckering, *Ens *enation

Studies to determine whether RLβ is trans-replicated by CRA and *vice versa*, agroinoculation experiments were conducted. Results suggested that RLβ could be transreplicated by CRA and plants co-infected with CRA and RLβ develop severe leaf curling, leaf crinkling, interveinal chlorosis, enation on underside of leaves and severe stunting (Table 4). Similarly, RLA could also trans-replicate with CRβ and this combination produce distinct symptoms in *N. benthamiana*. Therefore, both the DNAβs could be replicated by each other helper begomovirus leading to severe symptom phenotype (Table 4).

### RLA and CRA do not transreplicate DNA-B component of ToLCNDV and ToLCGV

**Figure 6:**
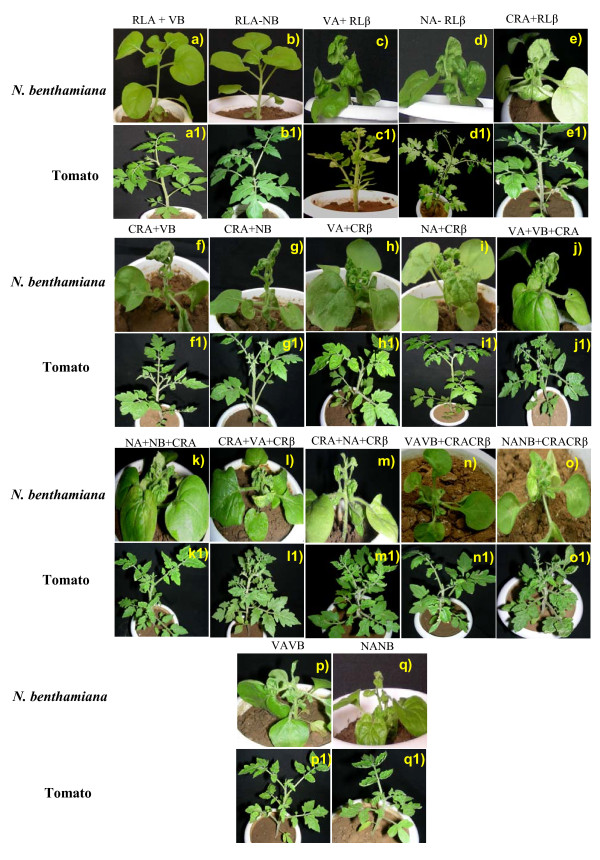
**Plant phenotypes following inoculation with radish and tomato-infecting begomoviruses**. Symptoms induced by the infectious virus constructs {RaLCV DNA-A (RLA), RaLCV DNA betasatellites (RLβ), CYVMV-[IN:Pat:Rad:08] DNA-A (CRA), CYVMV-[IN:Pat:Rad:08] betasatellites (CRβ), ToLCNDV DNA-A (NA), ToLCNDV DNA-B (NB) and ToLCGV DNA-A (VA), ToLCGV DNA-B (VB)} on tomato and *N. benthamiana *at 30 dpi, inoculated with different combinations (as labeled in each photograph).

Co-inoculation of RLA and CRA with either DNA-B of ToLCNDV (NB) or ToLCGV (VB) did not influence symptom severity nor the time required for symptom appearance (Figure 6a-a1, 6b-b1, f-f1 and 6g-g1; Table 4). Thus, the symptom expression pattern was similar as observed in plants inoculated with RLA and CRA alone. In addition, presence of DNA-B could not be ascertained for both the cases (Figure 4D-E and 5d). It is relevant to mention here that iteron sequences are different among RLA, CRA with both the DNA-B components of tomato-infecting begomoviruses. This could be a barrier for efficient transreplication of the heterologous DNA-B component.

### RLβ and CRβ can be a substitute for DNA B of either ToLCNDV or ToLCGV

ToLCNDV is a bipartite begomovirus that requires both DNA components for systemic infection [27]. Tomato plants inoculated with DNA-A (NA) and DNA-B (NB) of ToLCNDV developed leaf curling within 8 dpi and later the infected plants showed stunted growth along with severe leaf curling (30 dpi) (Figure 6q1), whereas tomato plants inoculated with only DNA-A remained symptomless (Table 4). Plants inoculated with NA alone lacked detectable levels of DNA in the newly emerged leaves when analyzed by PCR and Southern blot at 30 dpi (data not shown). To test whether a DNA betasatellite could influence the pattern of NA infection, tomato plants were inoculated with a mixture of NA + RLβ or NA + CRβ. A proportion (58%) of plants inoculated with NA + RLβ showed initial leaf curling (18 dpi) that later turned into pronounced leaf curling and enations at 30 dpi (Figure 6d1, Table 4). Southern blot hybridization confirmed the presence of replicative forms of both NA and RLβ in the distal leaves of symptomatic plants (Figure 4E lane 6 and G). However, accumulation of NA was lower in these plants as compared to plants inoculated with NA + NB (Figure 4G). Interestingly, NA + CRβ inoculated tomato plants remain symptomless and newly emerging leaves lacked detectable levels of either NA or CRβ (Figures 5b-c and 6i1.). *N. benthamiana *plants inoculated with NA + RLβ showed distinguishable symptoms like typical downward leaf curling, interveinal chlorosis and twisting of petiole (Figure 6d), whereas NA + CRβ induced leaf curling, twisting of petiole and leaf puckering (Figure 6i). Accumulation of viral DNA (NA) was less (~51%) in plants inoculated with NA + RLβ or NA + CRβ as compared to those inoculated with homologous combination (NA + NB) (Figures 4F and 5c). Hence, RLβ and CRβ could support efficient movement of NA in *N. benthamiana*. However, CRβ cannot facilitate efficient movement of NA on tomato unlike RLβ.

ToLCGV is a mono-bipartite species [28,29] and DNA-A (VA) alone is capable of causing systemic infection on *N. benthamiana *and tomato, although association of DNA-B results in increased symptom severity [28,29]. Neither RLβ nor CRβ could enhance systemic accumulation of VA as compared to VA + VB (Figures 4F-G, and 5e), although inoculated plants exhibited severe symptom (Table 4). For example, in *N. benthamiana*, presence of either RLβ or CRβ alongwith VA together caused twisting of the petiole, interveinal chlorosis and more stunting of the plants, while tomato plants exhibited stunting alongwith severe leaf curling as compared to plants inoculated with VA + VB or VA + CRβ (Figure 6c-c1, 6h-h1 and 6p-p1, Table 4).

### Synergism between CRA, ToLCNDV and ToLCGV

Since, CRA + CRβ could infect tomato and produced distinct symptoms like leaf curling, enation etc. (Table 4), we tested the potential consequences/interaction of these molecules with two predominant tomato-infecting begomoviruses (ToLCNDV and ToLCGV) from northern India. For this, tomato and *N. benthamiana *plants were inoculated with various combinations of viral DNA (Table 4). Plants inoculated with heterologous combinations of begomovirus species showed unusually severe symptoms as compared with plants infected with homologous combinations(CRA + CRβ or VA + VB or NA + NB) (Figure 6e-q). More interestingly, symptom severity was enhanced whenever plants were inoculated with CRA + CRβ alongwith either NA or VA. For example, when CRA or CRA + CRβ were mixed with VA or NA or VA + VB or NA + NB, plants exhibited severe symptoms (Figure 6j-o). Plants inoculated with both components of any two virus species exhibited symptoms at least a day earlier than those inoculated with CRA + CRβ (Table 4). To investigate synergism at the level of viral DNA accumulation, Southern blot analysis was conducted with probes specific for each of the six DNA components (Figure 5). The level of CRA in plants inoculated with CRA + VA + CRβ, CRA + NA + CRβ, CRA + VA + VB and CRA + NA + NB was low in tomato as compared to *N benthamiana *(Figure 5a). However, the level of CRA was ~1.4 times higher when VA + VB was also inoculated along with CRA + CRβ (Figure 5a), indicating CRβ assisted synergistic interaction. Unlike VA where no increase was observed, NA accumulation was marginally increased in plant inoculated with all four molecules (Figure 5c and 5e). Interestingly, level of either VB or NB remained unchanged in plants inoculated with any of the following combinations CRA + VA + VB, CRA + NA + NB, CRA + VA + VB + CRβ, CRA + NA + NB + CRβ as compared to either VA + VB or NA + NB, respectively (Figure 5d and 5f). The level of CRβ was reduced by 53% and 42% when it was co-inoculated with VA and NA, respectively as compared with the wild-type combination (Figure 5b). Accumulation of CRβ decreased upto 50-60% in plants inoculated with either CRA + VA + VB + CRβ or CRA + NA + NB + CRβ, in comparison to plants inoculated with CRA + CRβ alone (Figure 5b). Together, these results indicate that CRA may interact synergistically with VA + VB in the presence of CRβ.

## Discussion

In India, radish was not considered as a host for begomoviruses till Singh *et al. *[24] reported association of a begomovirus with leaf curl disease from Varanasi region. Subsequently, RaLCD was also observed from Pataudi, a place located ~800 km West from Varanasi. This indicates emergence of RaLCD as a new disease in India, incidence of which was recorded between 10-40%. Here, we ascertain that RaLCD is caused by a complex of two Old World monopartite begomovirus species and two distinct betasatellite molecules. On the basis of genome organization and phylogenetic analysis, the virus from Varanasi region (RaLCV-[IN:Var:05]) is considered as a novel begomovirus species, which share maximum sequence identity 87.7% with ToLCBV-[BD], whereas virus from Pataudi share maximum sequence identity 95% with CYVMV-IN and is considered as a CYVMV-IN variant on the basis of DNA-A sequence identity criteria of ICTV [2]. Generally, begomovirus isolates sharing less than 89% nucleotide sequence identity are considered to be distinct species, sharing 90-92% are considered as new strain and those with more than 94% identity and are considered to be variant of that strain [2]. Betasatellites associated with either RaLCV-[IN:Var:05] or CYVMV-[IN:Pat:Rad:08] was > 78% identical to TbLCB and CroYVMB, respectively, indicating they are isolates of previously described begomovirus associated betasatellite species [30]. Hence, betasatellites are designated as TbLCB-IN[IN:Var:Rad] and CroYVMB-[IN:Pat:Rad:08], respectively, which is also in concurrent with results of the phylogenetic analysis (Figure 2).

A current consensus regarding the extent of begomovirus diversity holds that high frequency of recombination, pseudorecombination and mutation is leading to emergence of highly pathogenic viruses which in turn cause economically important diseases [31-33]. The begomovirus causing RaLCD in the Pakistan can infect cotton and is strain of CLCuV [23]. However, in the present investigation, radish-infecting begomoviruses could not infect and produce symptom on cotton till 60 dpi. This is the first demonstration of Koch's postulates of radish-infecting begomoviruses, one of which is predominantly associated with infection on weed host, *Croton*. In the context of begomovirus emergence, it has been suggested that indigenous viruses infecting weed and wild hosts had been transferred to the new host, which facilitates emergence of novel species through recombination and/or pseudorecombination events [31-33]. Several new begomovirus species isolated and identified have arisen by recombination [28,34].

Recombination can provide selective advantage in the evolution of viruses at species, genera and family levels and has been documented in both animal and plant viruses with RNA or DNA genomes [35-37]. In this study, recombination was detected among full-length DNA-A component of one of these newly identified radish-infecting begomovirus species and it's near relatives by RDP analysis. The pattern of recombination and position of breakpoints is very convincing and supports earlier study about pattern of recombination in geminiviruses [32,38]. Breakpoint was detected in the virion sense ORF AV1/AV2, in RaLCV-[IN:Var:05] genome and for this region it shared maximum identity with PaLCuV. ORFs AC2 and AC3 of RaLCV-[IN:Var:05] also shares maximum identity for this region with PaLCuV. In the rep-coding region, recombination was found with a weed infecting begomovirus most similar to EuLCV. This region is commonly exchanged among begomoviruses [38] and may provide a selective advantage, such as enhanced replication or suppression of host defense. It is apparent that RaLCV-[IN:Var:05] is a chimeric molecule and has arisen by inter-specific recombination among EuLCV, PaLCuV and ToLCBDV. This result was also confirmed through Simplot analysis. Further, placement of RaLCV-[IN:Var:05] in a distinct phylogenetic tree with isolates of EuLCV, PaLCuV and ToLCBDV, monopartite begomoviruses from Asia supported this observation. These findings together indicate that recombination among begomoviruses is an important driving force in evolution of plant infecting begomoviruses with extending host range. An increasing number of monopartite begomoviruses has been reported in India in recent years, most of them are associated with DNA betasatellites molecules with evidences that some of them may have not evolved in the recent past [34].

Agroinoculation studies demonstrated that these monopartite viruses (RLA and CRA) can replicate autonomously and move systemically in their natural host (radish), and induce mild leaf curl symptoms. However, in the presence of DNA betasatellite, RLA or CRA induces these symptoms, which appear similar to those observed in the fields in Varanasi and Pataudi, India. Therefore, the RaLCD disease complex is caused by cognate association of DNA-A and DNA betasatellite. Prerequisite association of a begomovirus and satellite DNA-β has also been found to be necessary for typical disease development [14,15,34]. However, association of begomovirus and betasatelite complex with RaLCD has been demonstrated for the first time. Inability of RLA or CRA to transreplicate DNA-B molecule from either ToLCGV or ToLCNDV is attributed due to difference in iteron sequences and Rep binding sites observed. However, a number of recent studies have demonstrated that *Rep*-mediated binding can be more relaxed than thought previously [29,39]. Increase in symptom pattern and severity associated with betasatellites could be due to a direct effect of the βC1 protein on plant development [40,41]. Trans-complementation of RLβ and CRβ with CRA and RLA, respectively, revealed the promiscuous nature of betasatellite replication. This was further evidenced from the finding that two tomato-infecting begomovirus species (ToLCGV[IN:Var:01] and ToLCNDV[IN:Svr:93]) also served as helper viruses for RLβ and CRβ in *N. benthamiana *[42-45]. Our results also show that betasatellites complement the functions of the DNA-B component of tomato-infecting begomoviruses (ToLCGV and ToLCNDV), by mobilizing DNA-A from sites of inoculation to the distal tissues. These results fortify the idea that the βC1 protein can functionally substitute the role of DNA-B movement proteins [46].

Mixed infection of begomoviruses is common in Indian sub-continent and situation is further worsened owing to ability of whiteflies to transmit more than one begomovirus species under field conditions [47,48]. Since, *Croton yellow vein mosaic virus *and betasatellite DNA, isolated from RaLCD infected plant could infect tomato, we further extended study to test interaction of CRA and CRβ with predominant tomato-infecting begomoviruses species (ToLCNDV and ToLCGV) from India. Plants (*N. benthamiana *and tomato) inoculated with mixtures of either combinations of CRA + VA + CRβ, CRA + NA + CRβ, CRA + VA + VB, CRA + NA + NB, CRA + VA + VB + CRβ and CRA + NA + NB + CRβ, produced severe symptoms compared with plants infected with isolates of only one begomovirus species. Viral DNA accumulation (CRA) was also slightly enhanced in plants inoculated with CRA + VA + VB + CRβ or CRA + NA + NB + CRβ inoculated plants compared to plants inoculated with CRA + CRβ. Interestingly, beta satellite accumulation was decreased (upto 60%) in the presence of DNA-B component (either NB or VB) in the above combinations tested. This is in concurrent with earlier observation [49]. No significant increase of DNA accumulation of NA or VA was observed while increase of CRA in dual infected plants indicates asymmetric synergism (Figure 5). Asymmetric synergism has earlier been reported among two tomato-infecting begomoviruses [29].

## Conclusion

RaLCD in India is caused by association of two monopartite begomoviruses and promiscuous betasatellites. This is also the first experimental demonstration of Koch's postulate for begomoviruses associated with leaf curl disease of radish. The aetiology of this disease and the capacity of the DNA- betasatellite to be replicated by other begomovirus species increase the possibility of recombination and reassortment events. This could lead to evolution of new recombinant viruses or begomovirus complexes with different biological properties. It is also possible that exchange of betasatellites (RaLCV associated with TbLCB satellite DNA-β) could extend the virus host range thereby emergence of new diseases in cultivated crop plants (for example tomato, radish). The persistence of promiscuous betasatellites, such as RLβ and CRβ in radish or other hosts like weeds (e.g. *Croton *spp.) could facilitate the emergence of highly pathogenic begomovirus/betasatellite complexes that may induce more severe diseases by overcoming natural resistance of hitherto non-host.

## Methods

### Sample collection, grafting and DNA extraction

Surveys of radish-growing areas of Varanasi, Uttar Pradesh and Pataudi, Haryana in India were conducted and infected radish plants showing typical virus infection symptoms (stunted and distorted growth and leaf curl) were collected. The leaves alongwith petioles of at least 1-1/2 in. long were cut from the symptomatic plants and were used as scion. Infected scions were grafted onto the healthy radish leaf petiole, known as stock. Grafted plants were kept within an insect-proof glass house. Total nucleic acids were extracted from collected samples [50].

### Viral DNA detection and cloning

General detection of begomovirus association was carried out by Southern hybridization using coat protein probe [29] and by PCR with degenerate begomovirus specific primers [25]. PCR amplified DNA fragments were cloned into the pTZR57R/T vector (Fermentas Inc, USA) and sequenced. To facilitate full-length cloning of RaLCV-Varanasi genomic component (DNA-A), unique restriction site *Nde I *was determined, around which primer pairs RlNdeIFP (5'CATATGTGAGCCGTGTTG3') and RlNdeIRP (5'CATATGGGCTGTCGAAGT3') were designed.

For cloning of RaLCV-Pataudi, Haryana samples, viral DNA were amplified using Phi 29 DNA polymerase (Temphilphi; GE Healthcare) according to manufacturer's instruction. Briefly, the amplified concatemers were digested with restriction endonucleases to yield presumed monomeric virus components of ~2.8 kb. Potentially, full-length linearized viral genomes (at *Bam *HI site) were cloned into pKS^+ ^vector.

DNA-B degenerate primers [25] were used to amplify DNA-B component. The inability to amplify DNA-B using specific primer led us to design new set of primers corresponding to the common region shared by DNA-A and DNA-B followed by restriction endonuclease digestion of purified super-coiled DNA to identify two populations of molecules. Presence of betasatellite molecules was detected by PCR with primers beta01/02 [26]. Amplified products (~1.3 kb) were cloned into pTZR57R/T vector (Fermentas Inc, USA).

### Sequence comparisons

Putative full-length clones were purified using a Qiagen plasmid miniprep kit (Qiagen) and their sequences were determined commercially (Chromous Biotech, India). The sequence results were analyzed by comparing the sequence to other begomovirus sequences in GenBank using BLAST, followed by analysis using MEGA program version 4 (http://www.megasoftware.net/mega4/features.html) [51] with default parameters. The full-length genome sequences of representative begomoviruses and betasatellites were aligned using ClustalW and dendogram was generated using 1000 bootstrap repetitions. Standard abbreviations and GenBank accession numbers for DNA-A [2] and DNA betasatellites [30] were used.

### Recombination analysis

Detection of potential recombinant sequences, identification of likely parental sequences, and localization of recombination breakpoints were carried out with the recombination detection program (RDP version 3) [52]. Default RDP settings were used throughout with a P-value cutoff of 0.01 and the standard Bonferroni correction. For bootscan analysis, 200 replicates with a 95% cut-off were taken, and for GENECONV analysis, the g-scale parameter was set to 1. In addition, both the viral genome and DNA betasatellites sequences were analysed using Simplot program, version 3.2 (http://sray.med.som.jhmi.edu/SCRoftware/Simplot), with a sliding window of 200 nucleotides moving in 20-nucleotide steps [53-55].

### Generation of constructs for infectivity studies

To check infectivity, begomovirus and betasatellite were cloned as partial tandem repeat in pCAMBIA2301. For RaLCV-Var, ~1.6 kb *Bam *HI (152)-*Eco *RI (1789) fragment containing the intergenic region (IR) was cloned to generate a 0.6 mer (pRLAV0.6). The full-length monomer was cloned into the *Bam *HI digested pRLAV0.6 to generate a 1.6 mer tandem repeat called pRLAV1.6 (referred to here as RLA). For RaLCV-Patudi, a 2.4 kb *Bam *HI-*Hind *III fragment containing the IR was cloned to generate ~0.8 mer (pCRAP0.8). The full-length monomer was cloned into the *Bam *HI digested pCRAP0.8 to generate 1.8 mer tandem repeat called pCRAP1.8 (referred to here as CRA).

For the RaLCB-Var, *Pst I *(56)-*Kpn I *(1298) fragment was cloned into pCAMBIA2301, followed by ligation of full-length *Kpn *I fragment to produce partial tandem repeat called pRLβV1 (referred to here as RLβ). For the RaLCB-Pat, *Kpn *I (1292)-*Eco *RI (330) 395 bp fragment was released from RaLCB-Pat DNA-β and was cloned into pCAMBIA2301, followed by ligation of full-length *Kpn *I fragment to produce 1.3 mer tandem repeat called pCRβP1.3 (referred to here as CRβ). Insert orientation within partial tandem repeats were confirmed by restriction digestion with appropriate enzymes.

### Infectivity and trans-complementation of cloned DNAs

Recombinant plasmids (RLA, RLβ, CRA and CRβ) and the 'empty' vector (used as negative control) were introduced into *Agrobacterium tumefaciens *strain EHA105 by transformation. Agro-inoculation was performed in radish (cv Japanese White), *Nicotiana benthamiana *and tomato plants. Young seedlings (3-4 leaf stage) were inoculated viral constructs either alone or in combination by needle puncture method.

Trans-complementation with genomic components of tomato-infecting begomoviruses (*Tomato leaf curl New Delhi virus *[ToLCNDV] and *Tomato leaf curl Gujarat virus *[ToLCGV]) [32] was also studied. *Agrobacterium *cultures containing respective constructs (Table 4) were mixed in equal concentration and inoculated into test plants. The inoculated plants were placed in a controlled growth chamber with 16 h day light at 28°C/70% relative humidity. All the experiments were repeated at least for four times and symptom progression was recorded till 45 dpi.

### Detection of viral DNA

Total genomic DNA was extracted from leaf tissue at 30 dpi following Dellaporta method [50]. Viral and satellite DNAs was detected by PCR with the specific primers for RLA (RLF1.3 5'CTGGGCTTACCCATAGAGTGG3' & RLR1.3 5'CAGGGAAGACAATGTGGGCCT3'), CRA (CRF0.9 5'ATGGGTCTCTGCATATCCATGCCCTC3' & CRR0.9 5'TCAACTCGTCGACGCCTGATCCCTTTC3'), (RLβF 5'ATGACGATCAAATACAAAAACCAGAAAG3' RLβR 5'TTATACAGATGAACGCGTATACACATCG3') CRβ (CrβF 5'ATGACGATCATATATCAGAATGAGAC & CrβR 5'TTACACATTTACATATTTAGACACATC3'). In addition, specific primers were used for detection of genomic components of ToLCGV and ToLCNDV following Chakraborty *et al. *[29].

For southern hybridization analysis, total genomic DNA (8 μg) was separated on agarose gel (1%) and transferred to Hybond-N+ membrane (Amersham GE Healthcare, UK), following standard procedures [56]. Viral DNA was detected by hybridizing blots separately using radiolabelled probes of DNA-A and DNA- betasatellites specific to either species. For RLA, a *BseRI *fragment (nt 2097-2616) and for RLβ a *EcoRI *fragment (nt 299-506) was used as the probe. For CRA, a *Pst I*-*Xba I *(nt 1455-1884) fragment and for CRβ a *EcoRI*-*Nde I *fragment (nt 330-1041) were used. Specific probes for detection of DNA-A and DNA-B of ToLCNDV and ToLCGV were prepared according to Chakraborty *et al. *[29]. DNA fragments were labelled with [α-^32^P]dCTP by random oligonucleotide-primed synthesis [57]. Viral DNA levels were quantified using a PhosphorImager (Fuji Film).

## Competing interests

The authors declare that they have no competing interests.

## Authors' contributions

AKS performed the experiments. AKS, BC and SC were involved in data analysis experimental design and writing the manuscript. All the authors read and approved the final manuscript.

## References

[B1] Stanley J Bisaro DM Briddon RW Brown JK Fauquet CM Harrison BD Rybicki EP Stenger DC Fauquet CM, Mayo MA, Maniloff J, Desselberger U, Ball LA Geminivirida Virus Taxonomy: Eighth Report of the International Committee on Taxonomy of Viruses 2005 London: Elsevier/Academic Press 301 326

[B2] Fauquet CM Briddon RW Brown JK Moriones E Stanley J Zerbini M Zhou X Geminivirus strain demarcation and nomenclature Arch Virol 2008 153 783 821 10.1007/s00705-008-0037-6 18256781

[B3] Varma A Malathi VG Emerging geminivirus problems. A serious threat to crop production Ann Appl Biol 2003 142 145 164

[B4] Rojas MR Hagen C Lucas WJ Gibertson RL Exploiting chinks in the plant's armor: evolution and emergence of geminiviruses Annu Rev Phytopathol 2005 43 361 394 10.1146/annurev.phyto.43.040204.135939 16078889

[B5] Seal SE van den Bosch F Jeger MJ Factors influencing begomovirus evolution and their increasing global significance: implications for sustainable control Cri Rev Plant Sciences 2006 25 23 46

[B6] Lazarowitz SG Geminivirus: genome structure and gene function Crit Rev Plant Sci 1992 11 327 349

[B7] Laufs J Jupin I David C Schumacher S Heyraud-Nitschke F Gronenborn B Geminivirus replication: genetic and biochemical characterization of Rep protein function, a review Biochimie 1995 77 765 773 10.1016/0300-9084(96)88194-6 8824773

[B8] Sunter G Hartitz MD Hormudzi SG Brough CL Bisaro DM Genetic analysis of tomato golden mosaic virus: ORF AL2 is required for coat protein accumulation while ORF AL3 is necessary for efficient DNA replication Virology 1990 179 69 77 10.1016/0042-6822(90)90275-v 2219741

[B9] Stanley J Infectivity of the cloned geminivirus genome requires sequences from both DNA Nature 1983 305 643 645

[B10] Kheyr-Pour A Bendahmane M Matzeit V Accotto GPM Crespi S Gronenborn B *Tomato yellow leaf curl virus*: from Sardinia is a whitefly-transmitted monopartite geminivirus Nucleic Acids Res 1991 19 6763 6769 10.1093/nar/19.24.6763 PMC329307 1840676

[B11] Navot N Pichersky E Zeidan M Zamir D Czosnek H Tomato yellow leaf curl virus: a whitefly-transmitted geminivirus with a single genomic component Virology 1991 185 151 161 10.1016/0042-6822(91)90763-2 1926771

[B12] Dry IB Rigden JE Krake LR Mullineax PM Rezaian MA Nucleotide sequence and genome organisation of tomato leaf curl geminivirus J Gen Virol 1993 74 147 151 10.1099/0022-1317-74-1-147 8423446

[B13] Briddon RW Mansoor S Bedford ID Pinner MS Markham PG Clones of cotton leaf curl geminivirus induce symptoms atypical of cotton leaf curl disease Virus Genes 2000 20 17 24 10.1023/a:1008151921937 10766303

[B14] Briddon RW Mansoor S Bedford ID Pinner MS Saunders K Stanley J Zafar Y Malik K Markham PG Identification of DNA components required for induction of cotton leaf curl disease Virology 2001 285 234 243 10.1006/viro.2001.0949 11437658

[B15] Jose J Usha R Bhendi yellow vein mosaic disease in India is caused by association of a DNA β satellite with a begomovirus Virology 2003 305 310 317 10.1006/viro.2002.1768 12573576

[B16] Saunders K Norman A Gucciardo S Stanley J The DNA β satellite component associated with ageratum yellow vein disease encodes an essential pathogenicity protein (βC1) Virology 2004 324 37 47 10.1016/j.virol.2004.03.018 15183051

[B17] Cui X Tao X Xie Y Fauquet CM Zhou X A DNA β associated with tomato yellow leaf curl China virus is required for symptom induction J Virol 2004 78 13966 13974 10.1128/JVI.78.24.13966-13974.2004 PMC533896 15564504

[B18] Briddon RW Stanley J Subviral agents associated with plant single-stranded DNA viruses Virology 2006 344 198 210 10.1016/j.virol.2005.09.042 16364750

[B19] Kon T Rojas MR Abdourhamane IK Gilbertson RL Roles and interactions of begomoviruses and satellite DNAs associated with okra leaf curl disease in Mali, West Africa J Gen Virol 2009 90 1001 1013 10.1099/vir.0.008102-0 19264648

[B20] Saunders K Bedford ID Briddon RW Markham PG Wong SM Stanley J A unique virus complex causes ageratum yellow vein disease Proc Natl Acad Sci USA 2000 97 6890 6895 10.1073/pnas.97.12.6890 PMC18771 10841581

[B21] Briddon RW Bull SE Amin I Idris AM Mansoor S Bedford ID Dhawan P Rishi N Siwatch SS Abdel-Salam AM Brown JK Zafar Y Markham PG Diversity of DNA-β, a satellite molecule associated with some monopartite begomoviruses Virology 2003 312 106 121 10.1016/s0042-6822(03)00200-9 12890625

[B22] Zhou X Xie Y Tao X Zhang Z Li Z Fauquet CM Characterisation of DNA- associated with begomoviruses in China and evidence for co-evolution with their cognate viral DNA-A J Gen Virol 2003 84 237 247 10.1099/vir.0.18608-0 12533720

[B23] Mansoor S Mukhtar S Hussain M Amin I Zafar Y Malik KA Widespread occurrence of cotton leaf curl virus on radish in Pakistan Plant Dis 2000 84 80 10.1094/PDIS.2000.84.7.809B 30832124

[B24] Singh AK Chattopadhyay B Pandey PK Singh AK Chakraborty S A new Begomovirus species causing leaf curl disease of radish in India Plant Dis 2007 91 1053 10.1094/PDIS-91-8-1053B 30780448

[B25] Rojas MR Gilbertson RL Russel DR Maxwell DP Use of degenerate primers in the polymerase chain reaction to detect whitefly-transmitted geminiviruses Plant Dis 1993 77 340 347

[B26] Briddon RW Bull SE Mansoor S Amin I Markham PG Universal primers for the PCR-mediated amplification of DNA-β: a molecule associated with some monopartite begomoviruses Mol Bio-technol 2002 20 315 318 10.1385/MB:20:3:315 11936260

[B27] Padidam M Beachy RN Fauquet CM Tomato leaf curl geminivirus has bipartite genome and coat protein is not essential for infectivity J Gen Virol 1995 76 25 35 10.1099/0022-1317-76-1-25 7844539

[B28] Chakraborty S Pandey PK Banerjee MK Kalloo G Fauquet CM Tomato leaf Gujarat virus, a new begomovirus species causing a severe leaf curl disease of tomato in Varanasi, India Phytopathology 2003 93 1485 1496 10.1094/PHYTO.2003.93.12.1485 18943612

[B29] Chakraborty S Vanitharani R Chattopadhyay B Fauquet CM Supervirulent pseudorecombination and asymmetric synergism between genomic components of two distinct species of begomovirus associated with severe tomato leaf curl disease in India J Gen Virol 2008 89 818 828 10.1099/vir.0.82873-0 18272774

[B30] Briddon RW Brown JK Moriones E Stanley J Zerbini M Zhou X Fauquet CM Recommendations for the classification and nomenclature of the DNA-β satellites of begomoviruses Arch Virol 2008 153 763 781 10.1007/s00705-007-0013-6 18247103

[B31] Zhou X Liu Y Calvert L Munoz D Otim-Nape GW Robinson DJ Harrison BD Evidence that DNA-A of a geminivirus associated with severe cassava mosaic disease in Uganda has arisen by interspecific recombination J Gen Virol 1997 78 2101 2111 10.1099/0022-1317-78-8-2101 9267014

[B32] Padidam M Sawyer S Fauquet CM Possible emergence of new geminiviruses by frequent recombination Virology 1999 265 218 225 10.1006/viro.1999.0056 10600594

[B33] Pita JS Fondong VN Sangare A Otim-Nape GW Ogwal S Fauquet CM Recombination, pseudo-recombination and synergism of geminiviruses are determinant keys to the epidemic of the cassava mosaic disease in Uganda J Gen Virol 2001 82 655 661 10.1099/0022-1317-82-3-655 11172108

[B34] Kumari P Singh AK Sharma VK Chattopadhyay B Chakraborty S A novel recombinant tomato-infecting begomovirus capable of transcomplementing heterologous DNA-B components Arch Virol 2011 156 769 783 10.1007/s00705-011-0915-1 21311922

[B35] Domingo E Holland JJ RNA virus mutations and fitness for survival Annu Rev Microbiol 1997 51 151 178 10.1146/annurev.micro.51.1.151 9343347

[B36] Harrison BD Robinson DJ Natural genomic and antigenic variation in whitefly-transmitted geminiviruses (begomoviruses) Annu Rev Phytopathol 1999 37 369 398 10.1146/annurev.phyto.37.1.369 11701828

[B37] Garcı'a-Arenal F Fraile A Malpica JM Variability and genetic structure of plant virus populations Annu Rev Phytopathol 2001 39 157 186 10.1146/annurev.phyto.39.1.157 11701863

[B38] Lefeuvre P Lett JM Reynaud B Martin DP Avoidance of protein fold disruption in natural virus recombinants PLoS Pathog 2007 7 1782 1789 10.1371/journal.ppat.0030181 PMC2092379 18052529

[B39] Andrade EC Manhani GG Alfenas PF Calegario RF Fontes EPB Zerbini FM Tomato yellow spot virus, a tomato-infecting begomovirus from Brazil with a closer relationship to viruses from *Sida *sp, forms pseudorecombinants with begomoviruses from tomato but not from *Sida* J Gen Virol 2006 87 3687 3696 10.1099/vir.0.82279-0 17098986

[B40] Qazi J Amin I Mansoor S Iqbal MJ Briddon RW Contribution of the satellite encoded gene β C1 to cotton leaf curl disease symptoms Virus Res 2007 128 135 139 10.1016/j.virusres.2007.04.002 17482706

[B41] Yang JY Iwasaki M Machida C Machida Y Zhou X Chua NH β C1, the pathogenicity factor of TYLCCNV, interacts with AS1 to alter leaf development and suppress selective jasmonic acid responses Genes Dev 2008 22 2564 2577 10.1101/gad.1682208 PMC2546693 18794352

[B42] Mansoor S Briddon RW Bull SE Bedford ID Bashir A Hussain M Saeed M Zafar Y Malik KA Fauquet C Markham PG Cotton leaf curl disease is associated with multiple monopartite begomoviruses supported by single DNA-β Arch Virol 2003 148 1969 1986 10.1007/s00705-003-0149-y 14551819

[B43] Mansoor S Zafar Y Briddon RW Geminivirus disease complexes: the threat is spreading Trends Plant Sci 2006 11 209 212 10.1016/j.tplants.2006.03.003 16616578

[B44] Saunders K Briddon RW Stanley J Replication promiscuity of DNA-β satellites associated with monopartite begomoviruses; deletion mutagenesis of the Ageratum yellow vein virus DNA- β satellite localizes sequences involved in replication J Gen Virol 2008 89 3165 3172 10.1099/vir.0.2008/003848-0 19008407

[B45] Nawaz-ul-Rehman MS Mansoor S Briddon RW Fauquet CM Maintenance of an Old World betasatellite by a New World helper begomovirus and possible rapid adaptation of the betasatellite J Virol 2009 83 9347 9355 10.1128/JVI.00795-09 PMC2738271 19570867

[B46] Saeed M Zafar Y Randles JW Rezaian MA A monopartite begomovirus-associated DNA β satellite substitutes for the DNA B of a bipartite begomovirus to permit systemic infection J Gen Virol 2007 88 2881 2889 10.1099/vir.0.83049-0 17872543

[B47] Sanz AI Fraile A García-Arenal F Zhou X Robinson DJ Khalid S Butt T Harrison BD Multiple infection, recombination and genome relationships among begomovirus isolates found in cotton and other plants in Pakistan J Gen Virol 2000 81 1839 1849 10.1099/0022-1317-81-7-1839 10859391

[B48] Yang C Jia S Liu Z Cui G Xie L Wu Z Mixed Infection of Two Begomoviruses in Malvastrum coromandelianum in Fujian, China J Phytopathology 2008 156 553 555

[B49] Patil BL Fauquet CM Differential interaction between cassava mosaic geminiviruses and geminivirus satellites J Gen Virol 2010 91 1871 1882 10.1099/vir.0.019513-0 20335493

[B50] Dellaporta SL Wood J Hicks JB A plant DNA minipreparation. Version II Plant Mol Biol Rep 1983 1 19 21

[B51] Tamura K Dudley J Nei M Kumar S MEGA4: Molecular Evolutionary Genetics Analysis (MEGA) Software Version 4.0 Mo Biol Evol 2007 24 1596 1599 10.1093/molbev/msm092 17488738

[B52] Martin DP Williamson C Posada D RDP2: recombination detection and analysis from sequence alignments Bioinformatics 2005 21 260 262 10.1093/bioinformatics/bth490 15377507

[B53] Blawid R Van DT Maiss E Trans-replication of a Tomato yellow leaf curl Thailand virus DNA-B and replication of a DNA-β component by Tomato leaf curl Vietnam virus and Tomato yellow leaf curl Vietnam virus Virus Res 2008 136 107 117 10.1016/j.virusres.2008.04.025 18550192

[B54] Bouslama L Nasri D Chollet L Belguith K Bourlet T Aouni M Pozzetto B Pillet S Natural recombination event within the capsid genomic region leading to a chimeric strain of human enterovirus J Virol 2007 81 8944 8952 10.1128/JVI.00180-07 PMC1951430 17537864

[B55] Lole KS Bollinger RC Paranjape RS Gadkari D Kulkarni SS Novak NG Ingersoll R Sheppard HW Ray SC Full-length human immunodeficiency virus type 1 genomes from subtype C-infected seroconvertes in India, with evidence of intersubtype recombination J Virol 1999 73 152 160 10.1128/jvi.73.1.152-160.1999 PMC103818 9847317

[B56] Sambrook J Russell DW Molecular Cloning: a Laboratory Manual 2001 3 NY: Cold Spring Harbor, Cold Spring Harbor Laboratory

[B57] Feinberg AP Vogelstein B A technique for radiolabelling DNA restriction endonuclease fragments to high specific activity Ann Biochem 1983 132 6 13 10.1016/0003-2697(83)90418-9 6312838

